# A Comprehensive Experimental and Finite Element Analysis Study on the Bonding Strength Evaluation of Wafer-to-Wafer Hybrid Bonding with Polyimide Film Dielectrics

**DOI:** 10.3390/mi17050625

**Published:** 2026-05-19

**Authors:** Cong Mei, Tianze Zheng, Ziyang Ding, Dan Zhang, Yuan Xu, Huiyao Zhao, Liu Chang, Qiuhan Hu, Chenhui Xia, Shuli Liu, Liyi Li

**Affiliations:** 1 School of Integrated Circuits, Southeast University, Wuxi 214000, China; 230229587@seu.edu.cn (C.M.); 220236463@seu.edu.cn (T.Z.); 220246752@seu.edu.cn (Z.D.); 230250336@seu.edu.cn (D.Z.); xyuan12251004@163.com (Y.X.); kkkensmail@163.com (H.Z.); 230239483@seu.edu.cn (L.C.); 2 Technology Development & Central Laboratory, Shenzhen Kaifa Technology Co., Ltd., Shenzhen 518000, China; 3 School of Advanced Technology, Xi’an Jiaotong-Liverpool University, Suzhou 215123, China; qiuhan.hu23@student.xjtlu.edu.cn; 4 The 58th Research Institute of China Electronics Technology Group Corporation, Wuxi 214000, China; smartxvip@163.com (C.X.); liushuli_1990@126.com (S.L.)

**Keywords:** polyimide (PI) dielectric, hybrid bonding (HB), bonding strength, post-crack Di-cantilever bending (PBC-DCB), nanoindentation, finite element analysis (FEA)

## Abstract

Polymer insulation layers such as polyimide (PI) have gradually replaced inorganic dielectric layers (SiO_2_, SiCN) in the integrated packaging process of hybrid bonding (HB). PI can fill the gaps in the thermal compression bonding process and help to obtain a good Cu/Polymer bonding interface. At present, the existing post-crack double cantilever beam tensile test (PBC-DCB) has been successfully applied to the quantitative measurement of bonding strength of hybrid bonding with inorganic materials, but this method only considers elastic behavior. Since PI exhibits viscidity, elasticity and plasticity, knowing how to correlate these properties to the bonding process is challenging. Whether PBC-DCB is suitable for the characterization of PI bonding is unclear. This paper presents a comprehensive experimental and finite element analysis (FEA) study on the PI–PI bonding interface. Firstly, nanoindentation experiments and simulations are performed on the prepared PI interface to obtain key elasticity and plasticity parameters. Then, the bonding strength is characterized by the PBC-DCB test. Theoretical and experimental results show that the plasticity of PI causes energy dissipation during stretching, resulting in a deviation of approximately 2.51% compared with pure elasticity. Based on experimental data, the Cohesive Zone Model (CZM) FEA method is used to simulate the crack propagation. The results indicate that the Embedded Process Zone (EPZ) model can accurately describe crack initiation and delamination behavior, with a margin of error of about 3.61%. Finally, based on the EPZ CZM, defects such as bonding void and wafer warpage are further discussed in relation to bonding strength measurement.

## 1. Introduction

In recent years, considerable progress has been made in the research of hybrid bonding technology based on inorganic materials such as SiO_2_ and SiCN as dielectrics [1,2]. Some technologies have been applied from laboratory to the product (such as the stack of back-illuminated CMOS image sensors (BI-CIS) and NAND Flash) [3,4]. However, the hybrid bonding of PECVD SiO_2_ or SiCN imposes high requirements for the surface flatness, cleanliness and activation degree of the materials [5]. These limitations significantly increase manufacturing costs and reduce process output [6,7]. For the reasons mentioned above, polymer dielectric materials (polyimide (PI), poly-benzoxazole (PBO), and benzo cyclobutene (BCB)) have become strong competitors among the new generation of hybrid bonding and dielectric materials [8,9]. PI is easy to process and has a slight fluidity at high temperatures. During the bonding process, this fluidity can effectively fill the gaps in the thermal compression bonding process of the chip, which can improve the cavity problem, and help to obtain a good Cu/Polymer bonding interface [10]. In addition, polymers have high elongation, allowing them to effectively absorb mechanical stress and reduce warpage, thereby enhancing the reliability of heterogeneous integration [11]. However, the preparation and curing process of PI films involves solvent evaporation, imitation reaction and hydrophilic group bonding. Therefore, the PI film has a large adhesion area. So, determining the adhesion performance of the film is critical for predicting the HB process with polyimide film dielectrics.

Double cantilever bending (DCB) is a theoretical method that can accurately obtain bonding strength without measuring the crack length [12]. The precise initiation of bonding interface and water stress corrosion is hard to solve. As an improved PBC-DCB test method, a novel “post bonding crack” induced in the DCB method and measurements in a nitrogen atmosphere can suppress variations [13]. However, only elasticity is considered in the bonding strength calculation of inorganic materials. Even for the PI-Cu bonding interface, the plasticity of PI is still ignored [14]. How to evaluate the influence of the plastic performance of PI material on the strength of the bonded interface is a problem [15].

In this paper, a type of polyimide (PI) adhesive is applied to prepare PI films on a 12-inch Si wafer, and then the properties of PI films are characterized by nanoindentation experiments and FEA simulation. The test results reveal that PI films have typical elasticoplastic characteristics. To measure the bonding strength of the PI-PI interface, a PBC-DCB testing method is discussed. The test results reveal that when the bonding strength is very high, a crack will propagate to the substrate of silicon, leading to the force–displacement curve abnormality. When the bonding strength is reasonable, the PBC-DCB test method can be used to test the bonding strength. Theoretical and simulation studies show that the plasticity of PI will cause energy dissipation during stretching, resulting in a deviation of about 2.51% compared to pure elasticity. Based on experimental data, a Cohesive Zone Model (CZM) FEA method is used to simulate the crack propagation [13,16]. The results indicate that the Embedded Process Zone (EPZ) model can accurately describe crack initiation and delamination behavior. Finally, based on the EPZ CZM, defects such as bonding voids and wafer warpage are further discussed in relation to bonding strength measurement.

## 2. Nanoindentation Testing and Finite Element Simulation of PI Films

### 2.1. PI Film Preparation

**Figure 1:**
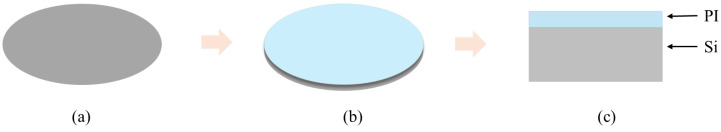
Schematic illustration of PI film preparation: (**a**) Si wafers’ cleaning and planarization; (**b**) PI spin coating and curing; (**c**) PI film treatment with CMP and activation process.

As shown in Figure 1, clean 12-inch bare 675 μm silicon (Si) wafers are used as substrates. PI varnish is coated by spin coater on the silicon wafers to achieve a cured film thickness of about 10 μm. Then, the wafers are pre-baked by UV exposure at 300 mJ/cm^2^ to 600 mJ/cm^2^. After UV exposure, the wafers should be cured at 200 °C for 2 h. After the curing is finished, PI films can be planarized to 5 μm by chemical mechanical polishing (CMP) and activated by plasma (O_2_).

### 2.2. Nanoindentation Testing

**Figure 2:**
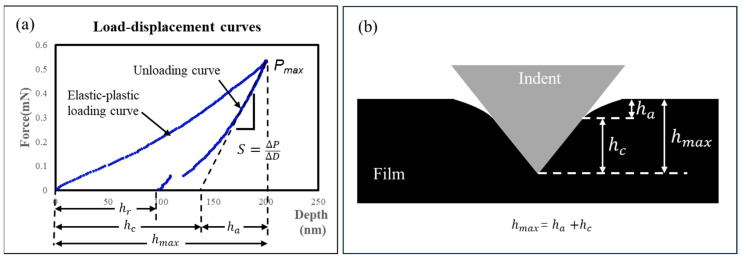
(**a**) Load-displacement curves of Quasi-Static Indentation testing for PI film; (**b**) schematic of contact between the indenter and PI film when the load depth is at maximum.

Nanoindentation technology has been widely applied as an important indicator for evaluating the mechanical behavior of various materials. When nanoindentation technology makes indentations on materials at the nanoscale, the Elastic Modulus and hardness of the sample material can be extracted through measurement results such as the load–displacement curve and contact stiffness obtained. This paper uses a Berkovich Indenter for testing. Since the PI-PI bonding interaction occurs at the interface, we utilized Quasi-Static Indentation to characterize the force–displacement behavior at a 200 nm depth, as shown in Figure 2a. A schematic showing the contact between the indenter and PI film is displayed in Figure 2b.

For a Berkovich diamond indenter, β is the indenter geometry shape factor, $E_{i}=1140\;{GP}_{a}$, $v_{i}=0.07$, $A_{c}=3\sqrt{3}h_{c}^{2}{tan}^{2}\theta ,\theta =65.27{}^{\circ}$.

From the principle of nanoindentation testing [17,18], the following can be known.

Contact stiffness:

(1) $$S=\frac{dP}{dh}=\frac{∆P}{∆D}$$

Reduced Modulus:

(2) $$E_{r}=\frac{S}{2\beta }\cdot \sqrt{\frac{\pi }{A_{c}}}=\frac{∆P}{∆D}\cdot \sqrt{\frac{\pi }{3\sqrt{3}h_{c}^{2}{tan}^{2}\theta }}=\frac{∆P}{∆D}\cdot \sqrt{\frac{\pi }{24.5\cdot h_{c}^{2}}}$$

Harness:

(3) $$H=\frac{P_{max}}{A_{c}}=\frac{P_{max}}{24.5\cdot h_{c}^{2}}$$

Elastic Modulus:

(4) $$E_{s}=\frac{1-v_{s}^{2}}{\frac{1}{E_{r}}-\frac{1-v_{i}^{2}}{E_{i}}}$$

Plasticity index [19]:

(5) $$\psi_{depth}=1-\frac{h_{max}-h_{r}}{h_{max}}=\frac{h_{r}}{h_{max}}$$

After many repeated tests, the results were as follows, as shown in Table 1.

**Figure 3:**
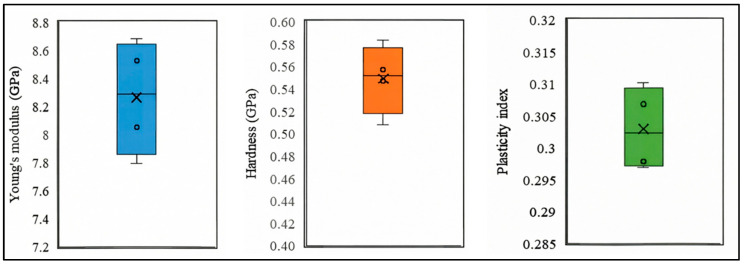
The Young’s Modulus, Harness and Plasticity index of PI film from nanoindentation.

From Table 1, it can be known that the Young’s Modulus of PI film is 8.264 GPa, the hardness is 0.549 GPa and $\psi_{depth}$ is 0.303. The error analysis is shown in Figure 3.

### 2.3. Nanoindentation Analytical Solution [20]

In the case of a Berkovich Indenter, an elastic–plastic response can be assumed. For the loading stage, the total depth can be found from the addition of contact depth and elastic depth as shown in Figure 2a.

The contact depth $h_{s1}$:

(6) $$h_{s1}={\left(\frac{P_{s}}{3\sqrt{3}H{tan}^{2}\theta }\right)}^{\frac{1}{2}}$$

(7) $${h_{s1}|}_{P_{s}=P_{max}}=h_{c}={\left(\frac{P_{max}}{3\sqrt{3}H{tan}^{2}\theta }\right)}^{\frac{1}{2}}$$

The elastic depth for unloading depth $h_{s2}$:

(8) $$h_{s2}=\varepsilon \frac{\sqrt{\pi P_{s}H}}{2E^{*}}$$

(9) $${h_{s2}|}_{P_{s}=P_{max}}=h_{a}=\varepsilon \frac{\sqrt{\pi P_{max}H}}{2E^{*}}$$

The total depth $h_{s}$:

(10) $$h_{s}=h_{s1}+h_{s2}={\left(\frac{P_{s}}{3\sqrt{3}H{tan}^{2}\theta }\right)}^{\frac{1}{2}}+\varepsilon \frac{\sqrt{\pi P_{s}H}}{2E^{*}}$$

(11) $${h_{s}|}_{P_{s}=P_{max}}=h_{max}=h_{s}+h_{a}={\left(\frac{P_{max}}{3\sqrt{3}H{tan}^{2}\theta }\right)}^{\frac{1}{2}}+\varepsilon \frac{\sqrt{\pi P_{max}H}}{2E^{*}}$$

For the unloading stage, the response of $h_{s}$ is elastic from $h_{max}$ to the partial unloading depth.

(12) $$h_{s}={\left(\frac{P_{s}}{P_{max}}\right)}^{\frac{1}{2}}\cdot \left(h_{max}-h_{r}\right)+h_{r}$$

where $h_{r}$ is the depth of residual impression, ε=0.75 for the Berkovich Indenter,

(13) $$h_{r}=\left(1-\frac{2}{\varepsilon }\right)h_{max}+\frac{2}{\varepsilon }h_{c}$$

**Figure 4:**
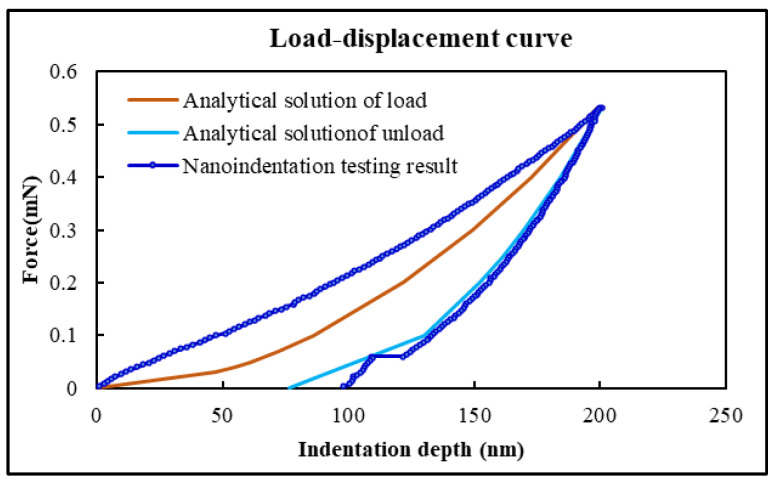
The load–displacement curves: contrast of analytical solution and nanoindentation testing results of PI film.

Assuming that the Young’s Modulus of PI film is 8.264 GPa, the hardness is 0.549 GPa, and the maximum indentation depth is 200 nm, its nanoindentation load–displacement curve can be simulated by Equations (9) and (11), which is shown in Figure 4.

Obviously, the analytical solution results are consistent with the nanoindentation testing result for the unloading stage. However, there are errors during the loading stage. This might be related to the planarization of the PI film surface. A larger roughness can easily lead to inaccurate zero-point positioning of the indent and a shift for the load–displacement curve.

### 2.4. Finite Element Analysis of Nanoindentation Testing

As the numerical approach developed, finite element analysis (FEA) was proposed for the simulation of load–displacement responses for PI film indentation testing [21]. We assume the contact between indentation and PI film is frictionless contact, and that the contact area is completely elasticity–plastic as embodied by Equations (9) and (10). For finite element modeling, nonlinear geometric behavior must be considered in the analysis.

**Figure 5:**
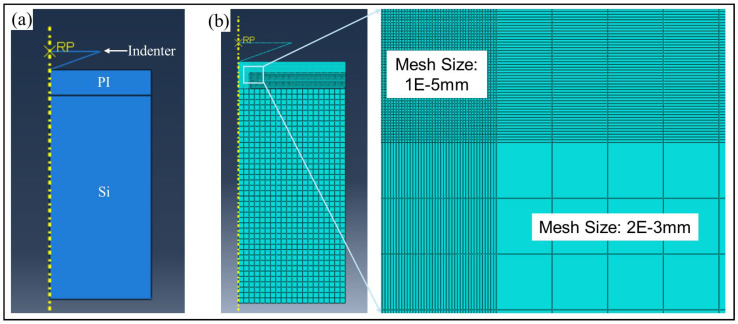
The FEA of PI indentation testing with Berkovich Indenter. (**a**) A 2D symmetric model with indentation, PI film and Si substrate. (**b**) The mesh setting of the FEA model.

A two-dimensional symmetric model as shown in Figure 5.

According to the FEA process, material parameters such as Young’s Modulus, Poisson’s ratio and yield stress are needed for further simulation. Referring to the indentation testing result, the elasticity modulus has been obtained as 8.264 GPa. Poisson’s ratio of PI film is set as 0.3. The yield is unknown and will be confirmed by FEA.

**Figure 6:**
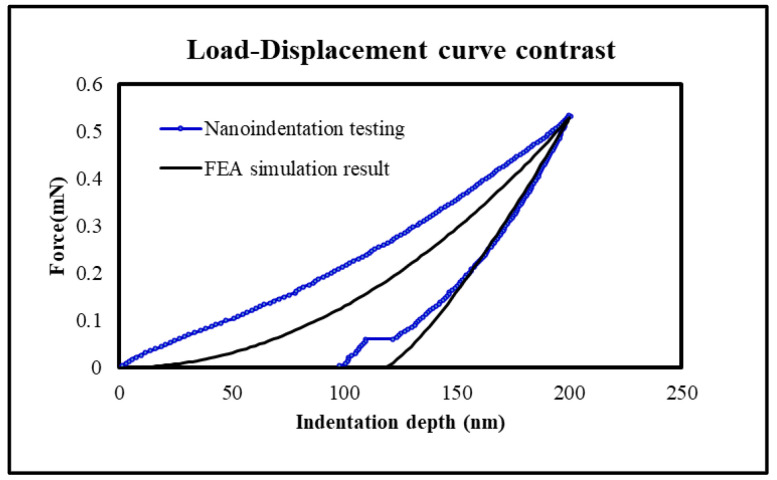
The contrast of load–displacement curves for the nanoindentation result and FEA simulation result.

Figure 6 shows the load–displacement when PI with yield stress is 0.458 GPa. It can be found that the curves of nanoindentation testing result and FEA simulation are nearly the same during the unloading stage.

**Figure 7:**
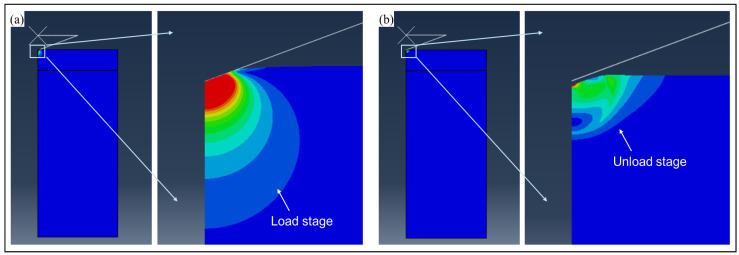
The strain diagram of PI film for indentation testing. (**a**) The PI film strain diagram when the load depth is $h_{max}$. (**b**) The residual strain during the unloading stage.

The strain diagram for the FEA simulation process is shown in Figure 7. As depicted, plastic areas can be found after the unloading stage.

Based on the above results, it can be known that the material parameters of PI film are shown in Table 2:

## 3. PBC-DCB Testing of PI Film Bonding

As an optimization method, the PBC-DCB method (a novel “post-bonding crack” introduced in the DCB method) has been successfully applied to quantitatively measure the bonding strength of linear elastic materials such as SiO_2_ and SiCN. This method can accurately obtain bonding strength without measuring the crack length at any position within the wafer. From the nanoindentation test, PI film has been confirmed to have typical elasticity and plasticity. The PI-PI bond strength will be discussed by examining the PBC-DCB test method in this section.

### 3.1. The Sample Preparation of PI-PI Bonding

**Figure 8:**
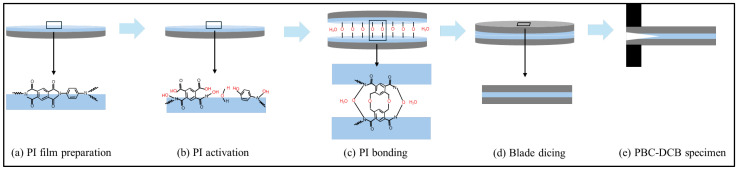
Hybrid bonding process workflow for PBC-DCB test specimen preparation. (**a**) PI film coating and curing; (**b**) PI film CMP and activation; (**c**) PI-PI bonding; (**d**) specimen dicing; (**e**) post-bonding crack fabrication and nut attachment.

The specimen for PBC-DCB tests is shown in Figure 8. After activation, sufficient hydrophilic groups (-OH) will form on the surface of PI. During the bonding stage, the hydrophilic groups will form bonding through dehydration. Cut the bonded wafers into test strips of 40 × 8 mm^2^. Finally, fix nuts to the front of sample with glue at room temperature.

### 3.2. PBC-DCB Model of PI-PI Bonding

**Figure 9:**
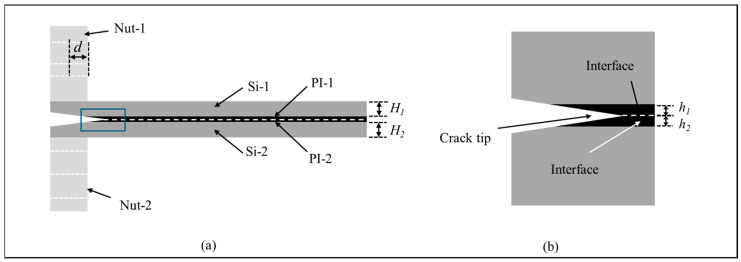
PBC-DCB testing method of PI-PI structure. (**a**) Side view of PBC-DCB structure, where the post-bending crack (PBC) is a trigonal prism; (**b**) magnification of crack tip located at the bonding interface.

The PBC-DCB model of PI-PI bonding is shown in Figure 9. The thickness of the silicon substrates and PI films are *H_i_* and *h_i_*, respectively. The index *i* = 1, 2 indicates the top and bottom wafers, respectively.

Based on Griffith’s elastic theory, the energy release rate during crack propagation is equal to the energy required to form a new crack surface.

(14) $$G_{c}=G_{e}=\frac{12P^{2}a^{2}}{Ebh^{3}}$$

When the crack propagates per unit area ∆A, assuming the work done by the system is ∆W, its energy release rate $G_{c}$ can be expressed as:

(15) $$G_{c}=\underset{∆A\to 0}{\lim}\left(\frac{∆W}{∆A}\right)$$

Considering the PBC-DCB test with elastic dielectrics, the energy release rate of the specimen can be obtained by the compliance derivative method:

(16) $$G_{c}=\frac{P^{2}}{2w}\cdot \frac{dC}{da}$$

And the compliance C and bonding strength $G_{c}$ can be expressed respectively as [12]:

(17) $$C=\frac{1}{E_{s}I_{2}{\lambda_{c}}^{3}}\left(\frac{{\lambda_{c}}^{3}\left(2a^{3}-2d^{3}\right)}{3}+2{a^{2}\lambda }_{c}^{2}+2ad\left(\lambda_{c}^{2}+\lambda_{c}\right)+d\lambda_{c}+1\right)+\frac{3\left(a-d\right)}{bG_{s}H}$$

(18) $$G_{c}=\frac{P^{2}}{2E_{s}I_{2}b{\lambda_{c}}^{3}}\left({{2a^{2}\lambda }_{c}}^{3}+4a\lambda_{c}^{2}+2d\left(\lambda_{c}^{2}+\lambda_{c}\right)\right)+\frac{3P^{2}}{2b^{2}G_{s}H}$$

When the bonding layer consists of elastic, plastic, and viscoelastic, Equation (13) should be corrected as:

(19) $$G_{s}=G_{e}+G_{P}+G_{v}$$

$G_{e}$, $G_{p}$, and $G_{v}$ are respectively the energy release rates of PI due to elastic strain, plastic strain and viscous strain during the tensile process when crack propagation occurs [22].

The total strain during the tensile process of PBC-DCB consists of elastic and plastic components and is expressed as:

(20) $$\varepsilon =\varepsilon_{e}+\varepsilon_{p}+\varepsilon_{v}$$

The viscous stress is expressed as:

(21) $$\sigma_{v}=\mu \cdot D_{v}$$

Here, μ is the shear modulus and $D_{v}$ is the strain rate. As shown, viscosity is a physical quantity related to the strain rate. Considering that the PBC-DCB testing process is a slow and steady stretching process and the viscosity of the cured PI is relatively low, the influence of viscosity can be temporarily ignored, $G_{v}=0$ [12].

For plasticity, the increment of plastic work done per unit volume of the material is:

(22) $$dW_{p}=\sigma_{p}\cdot d\varepsilon_{p}$$

The total plastic work for this plastic zone is:

(23) $$W_{p}=\int \int dW_{p}dA_{p}$$

**Figure 10:**
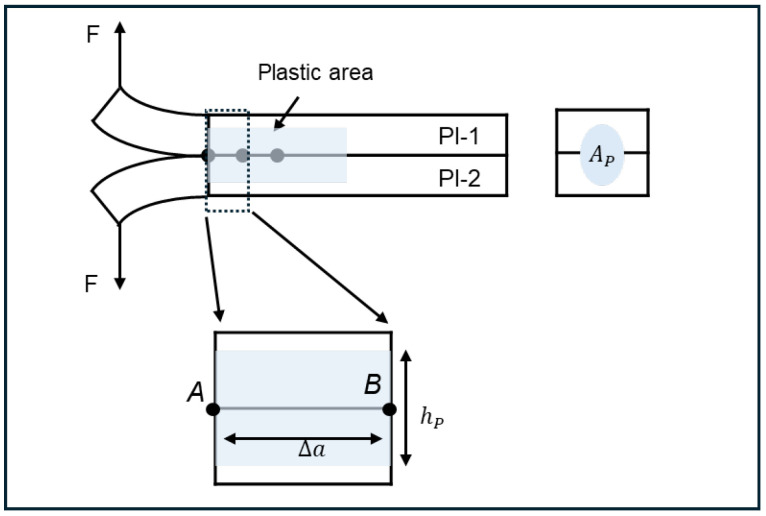
Schematic for calculations of plastic work.

The plastic zone size is independent of crack length since the specimen is under a steady state. If we assume that the crack growth is self-similar in terms of plastic zone shape and crack front geometry in the range of crack extension ∆a, the plastic work due to the crack extension can be expressed as:

(24) $$W_{∆a}=W_{p}\cdot h_{p}∆a$$

where $h_{p}$ is height of the plastic zone, and $h_{p}∆a$ is the area of plastically deformed material that is swept out by the crack extension ∆a. Then, the plastic component of the total energy, $G_{P}$ can be calculated from [22]:

(25) $$G_{p}=\frac{W_{∆a}}{∆V}=\frac{W_{p}h_{p}∆a}{A_{p}∆a}=\frac{W_{p}h_{p}}{A_{p}}$$

where the plastic energy $W_{p}$, plastic zone height $h_{p}$, and its area $A_{p}$ as shown in Figure 10.

Analytical solutions for $W_{p}$ are very hard to determine for different plastic zone areas. The elastoplastic finite element model is an effective way to obtain plastic energy.

The bonding strength can be calculated by:

(26) $$G_{c}=G_{s}=G_{e}+G_{P}$$

### 3.3. PBC-DCB Testing of PI-PI Bonding

As shown in Figure 8, the PI film will bond after activation. Two bonding conditions are discussed in this section. After temporary bonding, the coupons were permanently bonded under a nitrogen/hydrogen-mixed atmosphere at 1# and 2# conditions with an automated wafer bonding system (EVG-GEMINIFB), as shown in Table 3. The bonded interfaces were observed using scanning acoustic tomography (SAT). Table 4 displays the structure parameters of PBC-DCB specimen.

During PBC-DCB testing, the nuts are stretched with a constant displacement velocity of 0.01 mm/min in an atmosphere of pure N_2_ (moisture level < 1 ppm). The tensile tester has a load cell with a range of 0–200 N and an accuracy of ±0.02% of the full scale. The displacement measurement accuracy is ±0.5% of the displayed value. As displacement grows with a constant rate at blocks, the loading force is recorded by the tester. When noticing a drop in loading force, which means the crack has been propagated, the load is withdrawn so that beams can return to their initial position. These load–unload cycles are repeated until the specimen interface is completely separated.

**Figure 11:**
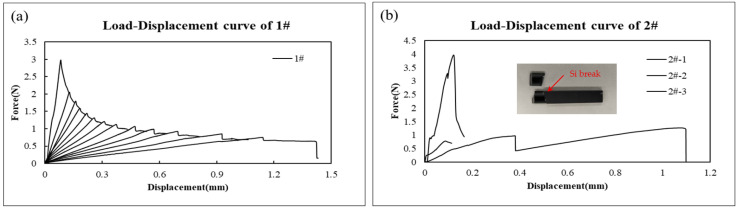
Load–displacement curves obtained from PBC-DCB testing. (**a**) L-D curve for 1#; (**b**) L-D curve for 2#-1, 2#-2, 2#-3.

The load–displacement (L-D) curves of 1# and 2# specimens are displayed in Figure 11. Test data show that 1# can achieve stable crack propagation during the tensile process of PBC-DCB specimen under condition 1 [22]. For the specimen bonding under condition 2, instability was observed during the testing, and fractures of 2# samples were found on the cantilever beam Si. Clearly, this is attributed to exceptionally high bond strength of the PI-PI interface under these conditions, which precludes accurate measurement via the PBC-DCB method.

**Figure 12:**
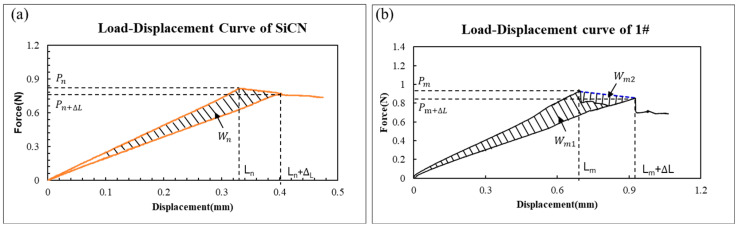
Load–displacement curve contrast for a crack extension in elastic–plastic materials and elastic–plastic materials. (**a**) Elastic materials (SiCN); (**b**) elastic–plastic materials (PI).

Figure 12 shows the typical load–displacement curve of elastic materials and elastic–plastic materials.

As shown in Figure 12a, assuming the initial crack length is a, when the load reaches $P_{n}$, the crack becomes critical and grows. Simultaneously, the load drops to $P_{n+∆L}$ and the crack propagation to a+∆a. The total energy dissipated in the crack growing process of ∆a is the shaded area ${∆E}_{n}$.

(27) $${{∆E}_{n}=W}_{n}=\frac{1}{2}\left[{P_{n}}^{2}C\left(L_{n}\right)-{P_{n+∆L}}^{2}C\left(L_{n}+∆L\right)\right]$$

The total energy release rate:

(28) $$G_{c}=\frac{{∆E}_{n}}{w∆a}$$

As shown in Figure 12b, referring to Equation (20), the total energy for the crack growing process is the shaded area ${∆E}_{m}$.

(29) $${{∆E}_{m}=W}_{m1}=\frac{1}{2}\left[{P_{m}}^{2}C\left(L_{m}\right)-{P_{m+∆L}}^{2}C\left(L_{m}+∆L\right)\right]-W_{m2}=W_{m}-W_{m2}$$

The total energy release rate:

(30) $$G_{c}=G_{m1}=\frac{{∆E}_{m}}{w∆a}=\frac{W_{m1}}{w∆a}=\frac{W_{m}-W_{m2}}{w∆a}=G_{m}-G_{m2}$$

The plastic dissipation energy release:

(31) $$G_{m2}=\frac{W_{m2}}{w∆a}$$

**Figure 13:**
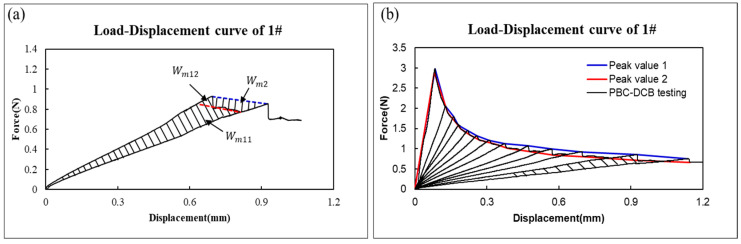
(**a**) Load–displacement curve of PI film with crack propagation ∆a; (**b**) Peak 1 and Peak 2 are introduced to the load–displacement curve of PI film for contrast.

Referring to Equation (22) and Figure 13a, the bonding strength can also be estimated as:

(32) $$G_{c}=G_{e}+G_{p}=\frac{{∆E}_{m}}{w∆a}=\frac{W_{m1}}{w∆a}=\frac{W_{m11}+W_{m12}}{w∆a}=G_{c11}+G_{c12}$$

where

The elastic energy release rate is $G_{e}=G_{m11}=\frac{W_{m11}}{w∆a}=G_{c11}$;

The plastic energy release rate is $G_{p}=G_{m12}=\frac{W_{m12}}{w∆a}=G_{c12}$;

To calculate the bonding strength, $G_{c1}$ and $G_{c2}$ are introduced, as shown in Figure 13b. Clearly, $G_{c1}=G_{m}$, $G_{c2}=G_{m11}$.

(33) $$G_{c2}<G_{c}<G_{c1}$$

where $G_{c2}$ can be calculated by peak value 2 and $G_{c1}$ can be calculated by peak value 1 as shown in Figure 13b.

**Figure 14:**
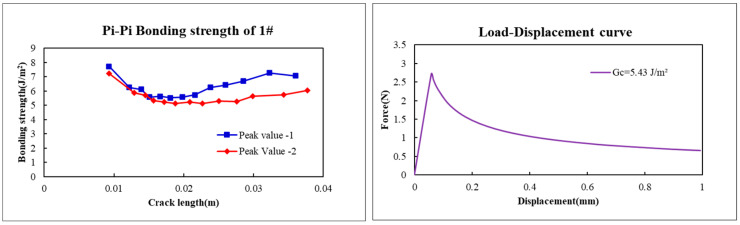
Bonding strength calculated from Peak value 1 and Peak value 2 by Equations (16) and (17).

The bonding strength of 1# can be confirmed as being between 5.29~5.57 J/m^2^, as shown in Figure 14.

For simplicity, assuming the plastic depth is less than 200 nm and the plastic depth index $\psi_{depth}$ is 0.303 (calculated by Equation (5)), the $G_{s}=G_{c2}$.

The energy release rate:

(34) $$G_{c}=G_{m}-G_{m2}=\frac{12P^{2}a^{2}}{Ebh^{3}}-\frac{12P^{2}a^{2}}{Eb{\left(h{\cdot \psi }_{depth}\right)}^{3}}=\left(1-\frac{1}{\psi_{depth}^{3}}\right)\cdot G_{m}$$

Then, when $G_{c}=5.43\;J/m^{2}$, the deviation is 2.51% compared with pure-elasticity bonding strength $\left(5.57\;J/m^{2}\right)$.

## 4. Finite Element Analysis of PI-PI PBC-DCB Testing

For the PBC-DCB structure of elastic–plastic model, it is very difficult to obtain its analytical solution for the structural complexity brought about by its plastic deformation, referring to Equations (18)–(25). The development of modern computer technology and numerical calculation methods has provided a new method for the study of mechanical properties of bonded components. At present, many studies based on PI materials use the Cohesive Zone Model (CZM) law of bilinear model to predict crack initiation and interface delamination behavior, and the plasticity of PI materials is ignored in the simulation process.

### 4.1. Cohesive Zone Model (CZM) Law

**Figure 15:**
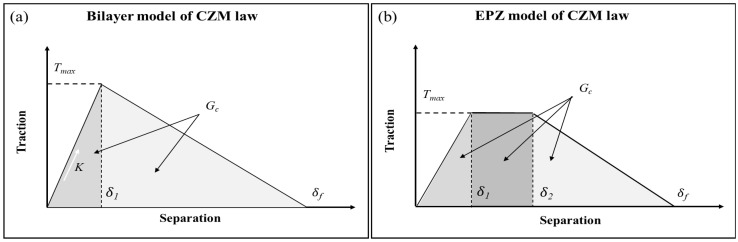
Two T-S relationship curves of CZM laws. (**a**) Bilayer model; (**b**) EPZ model.

The CZM is established based on the cohesion stress-separation displacement relationship (traction separation, abbreviated as T-S relationship) at the upper and lower ends of the interface. It has advantages in describing material fracture and can effectively characterize the damage failure process of the bonding layer under loading conditions. Here are two T-S relationship curves introduced in Figure 15 [23,24].

Figure 15a shows the traction separation (T-S) law of the bilinear CZM under tension. The index “1” and “f” mean the damage initiation and damage completion at the interface. The displacement of the interface damage initiation and complete damage can be represented by $\delta_{1}$ and $\delta_{f}$, and the normal forces of interface damage initiation is denoted by *T_max_*. Both the upward and downward sections of the T-S curve are linear. The bilinear CZM is relatively simple in form and easy to implement by the finite element method.

In addition, when only opening mode crack is considered, the interface fractured energy $G_{c}$ can be expressed as:

(35) $$G_{c}=\frac{\delta_{f}\cdot T_{max}}{2}$$

(36) $$K=\frac{T_{max}}{\delta_{o}}$$

Figure 15b shows the traction separation (T-S) law of the Embedded Process Zone (EPZ) CZM. This model divides the interface constitutive relationship into three stages: linear elasticity, ideal plasticity, and linear softening. The index “1”, “2”and “f” mean the damage initiation, plasticity stage and damage completion at the interface. The displacement of the interface damage initiation and complete damage can be represented by $\delta_{1}$ and $\delta_{f}$, and the normal forces of interface damage initiation are denoted by *T_max_*.

The interface fractured energy $G_{c}$ can be expressed as [25,26]:

(37) $$G_{c}=\frac{1}{2}T_{max}(\delta_{f}+(\delta_{2}-\delta_{1}))$$

**Figure 16:**
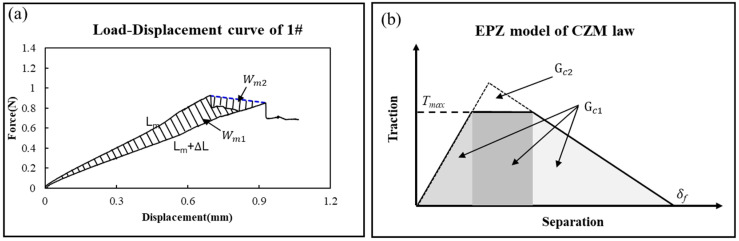
The crack propagation of L-D curve and EPZ model explaining (**a**) the energy dissipation because of the PI plasticity and (**b**) the bonding strength decrease because of PI plasticity.

Because of the elasticity and plasticity of PI film, the EPZ model will be used in the following FEA discussions, as shown in Figure 16. For the EPZ model of the CZM law, some parameters (i.e., $G_{c}$, $E_{s}$ and Y) are determined from nanoindentation and PBC-DCB tests, while the rest of the parameters (i.e., *T_max_*, $\delta_{1}$, $\delta_{2}$ and $\delta_{f}$ as) can be obtained by simulating PBC-DCB tests and then matching the load–displacement curves derived from the simulations with the experiment.

### 4.2. Finite Element Model of PBC-DCB Test

**Figure 17:**
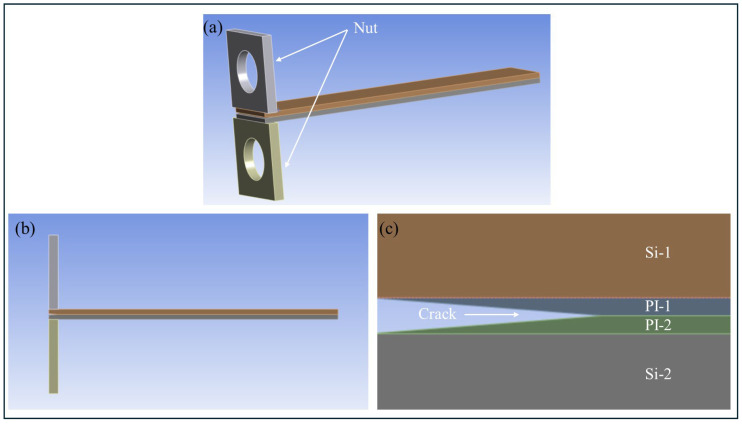
Geometry of PBC-DCB test structure. (**a**) 3D view; (**b**) side view; (**c**) magnification of PBC.

Referring to Figure 8, a finite element model is established as shown in Figure 17.

**Figure 18:**
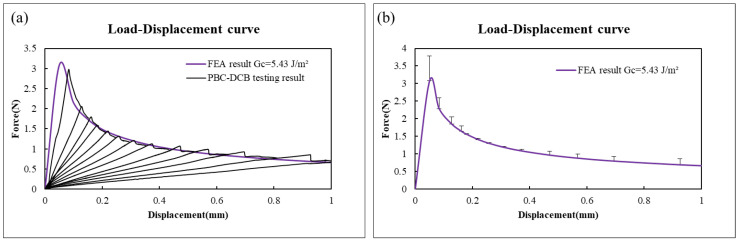
Comparison of the FEA simulation results with PBC-DCB testing result. (**a**) Load–displacement curves contrast. (**b**) Error bar analysis; the average is 3.61%.

Figure 18 shows the comparison of the FEA simulation results with PBC-DCB testing result, and the error bar is about 3.61%. The EPZ model parameter of PBC-DCB testing for PI-PI bonding is shown in Table 5.

### 4.3. FEA of PBC-DCB Specimen with Bonding Void

Void of the bonding interface is an inevitable problem for the hybrid bonding process. To simplify the research, void is temporarily ignored in both the samples’ bond strength testing and the finite element model simulation. This section will discuss the influence of interface voids on bond strength testing by the FEA methods.

**Figure 19:**
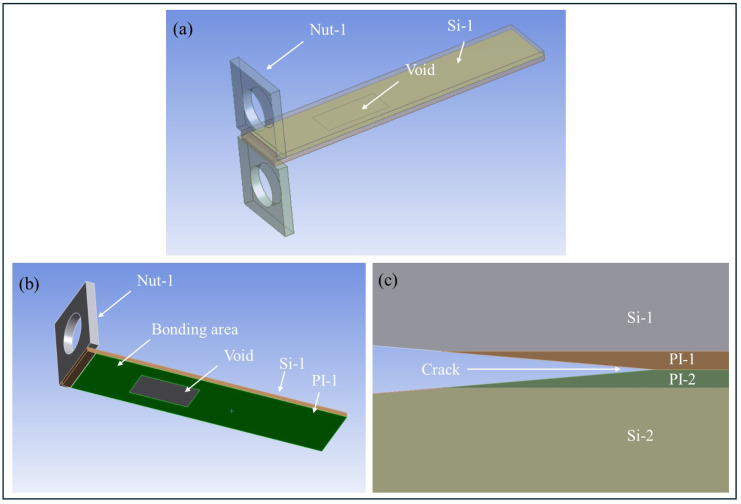
Geometry of PBC-DCB with a bonding void. (**a**) 3D view; (**b**) interface of PI-PI bonding. (**c**) Magnification of PBC.

Based on the above EPZ model of CZM law, the influence of void can be discussed. The finite element model is shown in Figure 19; bonding void is added to the interface.

**Figure 20:**
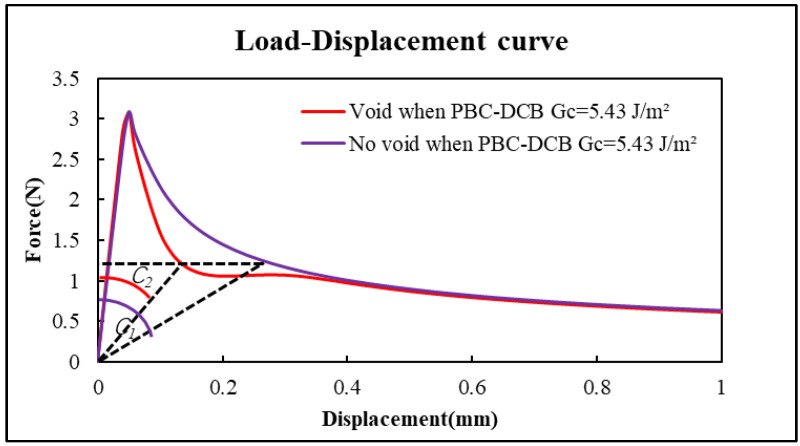
The comparison of load–displacement curves for the FEA results. Purple curve: PBC-DCB without void. Red curve: PBC-DCB with void.

As shown in Figure 20, when the bonding interface has a void, the load–displacement compliance $C_{2}$ of PI-PI with a void is less than the $C_{1}$ of PBC-DCB sample without a void.

(38) $$C=\frac{D}{P}$$

(39) $$C_{2}<C_{1}$$

Therefore, when the bonding interface of PBC-DCB specimen has a void, the bonding strength will be lower if using Equations (17) and (18) to perform calculations.

(40) $$G_{c2}<G_{c1}$$

### 4.4. FEA of PBC-DCB Specimen with Warpage

**Figure 21:**
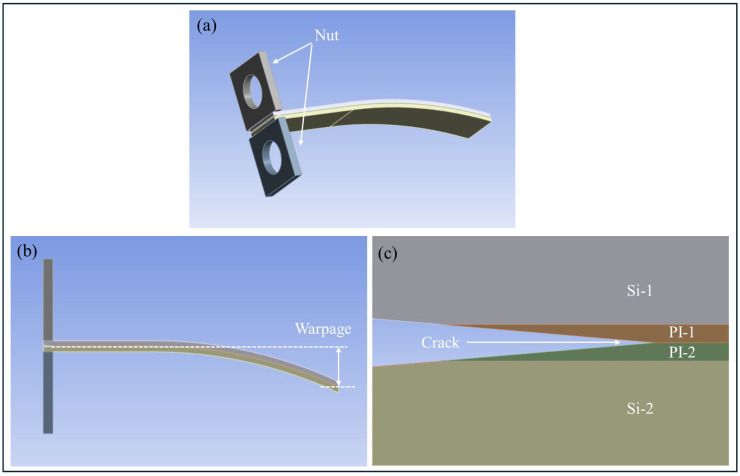
Geometry of PBC-DCB with warpage. (**a**) 3D view; (**b**) side view of the warpage. (**c**) Magnification of PBC.

Wafer warpage is an inevitable problem after hybrid bonding. For model simplification and theoretical analysis, the influence of wafer warpage is ignored during the PBC-DCB test and FEA. Figure 21 shows the 3D geometry of PBC-DCB when it has warpage of 5 mm.

Assuming the bonding strength $G_{c}=5.43$ J/m^2^, the load–displacement curves of the sample with warpage and without warpage were compared in Figure 21.

Clearly, the compliance $C_{2}$ of PI-PI with warpage is less than the $C_{1}$ of PBC-DCB sample without warpage.

(41) $$C_{2}<C_{1}$$

Therefore, when the bonding interface of PBC-DCB specimen has warpage, the bonding strength will be lower if using Equations (17) and (18) to perform calculations.

**Figure 22:**
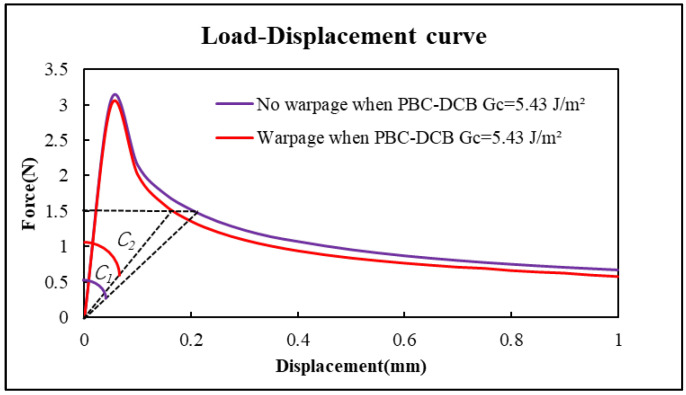
The comparison of load–displacement curves for the FEA results. Purple curve: PBC-DCB without warpage. Red curve: PBC-DCB with warpage.

The comparison of load–displacement curves for the FEA results are shown in Figure 22. Purple curve: PBC-DCB without warpage. Red curve: PBC-DCB with warpage.

## 5. Conclusions

In this paper, a comprehensive experimental and FEA study were proposed to analyse the bonding strength of wafer-to-wafer hybrid bonding with polyimide film dielectrics. The main conclusions are summarized as follows:

(1) By nanoindentation and FEA, polyimide (PI) exhibits obvious elastoplastic mechanical behavior, which is significantly different from inorganic dielectric materials (SiO_2_, SiCN) that follow linear elasticity. The Young ‘s Modulus and yield stress of prepared PI film are 8.076 GPa and 0.458 GPa, respectively.
(2) A PBC-DCB test method is proposed to characterize the bonding strength of PI-PI. Theoretical and experimental results show that the plasticity of PI causes energy dissipation during stretching, resulting in a deviation of approximately 2.51% when compared with pure-elasticity bonding strength.
(3) Based on experimental data, a Cohesive Zone Model (CZM) FEA method is used to simulate crack propagation. The results indicate that the EPZ model can accurately describe crack initiation and delamination behavior. Finally, defects from factors such as bonding voids and wafer warpage were further discussed in relation to the bonding strength measurement. Void and warpage will lead to lower testing results.

This research provides key support for material selection, process optimization, and yield improvement of hybrid bonding technology in the heterogeneous integration of AI chips. We will focus on bonding strength measurement for PI/Cu g in the future.

## Figures and Tables

**Table 1:** The indentation testing results of PI film.

No.	Max Depth (nm)	Plastic Depth (nm)	Max Load (mN)	Hardness (GPa)	Er (GPa)	Es (GPa)	$\psi_{depth}$
1	200.563	140.836	0.531	0.547	9.371	8.52791	0.298
2	201.282	141.538	0.521	0.557	9.544	8.684681	0.297
3	201.346	138.887	0.535	0.508	8.5634	7.792911	0.310
4	201.872	139.936	0.525	0.583	8.847	8.050826	0.307

**Table 2:** The material parameters of PI film.

Material Parameters of PI Film	Value
Young’s Modulus (GPa)	8.264
Poisson’s ratio	0.3
Hardness (GPa)	0.549
Yield stress (GPa)	0.458

**Table 3:** The PI film bonding condition.

No.	Bonding Condition	Annealing
1#	150 °C, 2.5 MPa and 5 min	60 S
2#	250 °C, 8.0 MPa and 5 min	60 S

**Table 4:** PBC-DCB parameters of PI-PI bonding.

Parameter	Symbol (Unit)	Value
Young’s Modulus of Si	*E_s_* (GPa)	166
Poisson’s ratio of Si	*v_s_*	0.28
Young’s Modulus of PI	*E_c_* (GPa)	8.264
Poisson’s ratio of PI	v_c_	0.3
Yield of PI	*Y* (GPa)	0.549
The thickness of Si-1	*H*_1_ (mm)	0.675
The thickness of Si-2	*H*_2_ (mm)	0.675
The thickness of PI-1	*h*_1_ (mm)	0.005
The thickness of PI-2	*h*_2_ (mm)	0.005
The width of wafer	*w* (mm)	8
Half-length of nut	*d* (mm)	0.65

**Table 5:** EPZ model of PBC-DCB testing for PI-PI bonding.

Parameter	Symbol (Unit)	Value
Bonding strength (J/m^2^)	$G_{c1}$	5.43
Separation 1 (mm)	$\delta_{1}$	0.0046
Separation 2 (mm)	$\delta_{2}$	0.0052
Normal Separation Across the Interface (mm)	$\delta_{f}$	0.023
Maximum Normal Traction (MPa)	*T_max_*	0.5

## Data Availability

The data are included in the article. If further information is required, the data are available upon request from the authors.
